# ALDH2 attenuates myocardial pyroptosis through breaking down Mitochondrion-NLRP3 inflammasome pathway in septic shock

**DOI:** 10.3389/fphar.2023.1125866

**Published:** 2023-03-13

**Authors:** Ying Zhang, Ying Lv, Qingju Zhang, Xingfang Wang, Qi Han, Yan Liang, Simeng He, Qiuhuan Yuan, Jiaqi Zheng, Changchang Xu, Xiangxin Zhang, Zichen Wang, Huaxiang Yu, Li Xue, Jiali Wang, Feng Xu, Jiaojiao Pang, Yuguo Chen

**Affiliations:** ^1^ Department of Emergency Medicine, Qilu Hospital of Shandong University, Jinan, China; ^2^ Chest Pain Center, Shandong Provincial Clinical Research Center for Emergency and Critical Care Medicine, Institute of Emergency and Critical Care Medicine of Shandong University, Qilu Hospital of Shandong University, Jinan, China; ^3^ Key Laboratory of Emergency and Critical Care Medicine of Shandong Province, Key Laboratory of Cardiopulmonary-Cerebral Resuscitation Research of Shandong Province, Shandong Provincial Engineering Laboratory for Emergency and Critical Care Medicine, Qilu Hospital of Shandong University, Jinan, China; ^4^ The Key Laboratory of Cardiovascular Remodeling and Function Research, Chinese Ministry of Education, Chinese Ministry of Health and Chinese Academy of Medical Sciences, The State and Shandong Province Joint Key Laboratory of Translational Cardiovascular Medicine, Qilu Hospital of Shandong University, Jinan, China

**Keywords:** septic shock, aldehyde dehydrogenase 2, myocardial pyroptosis, NLRP3 inflammasome, HADHA

## Abstract

Cell survival or death is critical for cardiac function. Myocardial pyroptosis, as a newly recognized programmed cell death, remains poorly understood in sepsis. In this study, we evaluated the effect of aldehyde dehydrogenase (ALDH2) on myocardial pyroptosis and revealed the underlying mechanisms in sepsis. We established a septic shock mice model by intraperitoneal injection of Lipopolysaccharide (LPS, 15 mg/kg) 12 h before sacrifice. It was found that aldehyde dehydrogenase significantly inhibited NOD-like receptor protein 3 (NLRP3) inflammasome activation and Caspase-1/GSDMD-dependent pyroptosis, which remarkably improved survival rate and septic shock-induced cardiac dysfunction, relative to the control group. While aldehyde dehydrogenase knockout or knockdown significantly aggravated these phenomena. Intriguingly, we found that aldehyde dehydrogenase inhibited LPS-induced deacetylation of Hydroxyacyl-CoA dehydrogenase trifunctional multienzyme complex α subunit (HADHA) by suppressing the translocation of Histone deacetylase 3 (HDAC3) from nuclei to mitochondria. Acetylated HADHA is essential for mitochondrial fatty acid β-oxidation, and its interruption can result in accumulation of toxic lipids, induce mROS and cause mtDNA and ox-mtDNA release. Our results confirmed the role of Histone deacetylase 3 and HADHA in NOD-like receptor protein 3 inflammasome activation. *Hdac3* knockdown remarkedly suppressed NOD-like receptor protein 3 inflammasome and pyroptosis, but *Hadha* knockdown eliminated the effect. aldehyde dehydrogenase inhibited the translocation of Histone deacetylase 3, protected ac-HADHA from deacetylation, and significantly reduced the accumulation of toxic aldehyde, and inhibited mROS and ox-mtDNA, thereby avoided NOD-like receptor protein 3 inflammasome activation and pyroptosis. This study provided a novel mechanism of myocardial pyroptosis through mitochondrial Histone deacetylase 3/HADHA- NOD-like receptor protein 3 inflammasome pathway and demonstrated a significant role of aldehyde dehydrogenase as a therapeutic target for myocardial pyroptosis in sepsis.

## Introduction

Sepsis is a life-threatening condition characterized by organ dysfunction due to unregulated host immune response against infection (Singer et al., 2016), and is widely recognized as the ultimate cause of death from many diseases. Nearly 50 million cases of sepsis were reported worldwide in the year of 2017 (Kempker and Martin, 2020; Rudd et al., 2020), and this number was considered significantly underestimated. More than 60% of patients with severe sepsis or septic shock were reported to have cardiac dysfunction (Pulido et al., 2012), the presence of which is related to mortality as high as 70%–90% (Parrillo et al., 1990; Merx and Weber, 2007; Beesley et al., 2018; Hollenberg and Singer, 2021). Therefore, exploring novel therapeutic targets for septic shock-induced cardiac dysfunction is a key research imperative.

Programmed death of cardiomyocytes is one of the critical mechanisms of impaired cardiac function (Del Re et al., 2019). In the last two decades, several types of programmed cell death were newly recognized and interpreted, such as necroptosis, ferroptosis and pyroptosis. Of all the types of cell death, pyroptosis is most closely related to infection and inflammation. It is also referred as Gasdermin-dependent inflammatory programmed cell death, and characterized by rapid rupture of cell membrane and the release of inflammatory cytokines and cellular contents (Bergsbaken et al., 2009; Li et al., 2019). Thus, pyroptosis can be triggered by inflammation and further greatly amplifies the inflammation response, which may play key role in septic shock-induced cardiac dysfunction. NOD-like receptor protein 3 (NLRP3) inflammasome/Caspase-1/Gasdermin D (GSDMD) pathway is the well-known canonical regulatory pathway of pyroptosis. NRLP3 inflammasome is a multi-protein complex composed of intracellular receptor NLRP3, adaptor protein apoptosis-associated speck-like protein (ASC) and precursor pro-Caspase-1 (Swanson et al., 2019; Xue et al., 2019). The activation of this complex ultimately cleaves pro-Caspase-1 to its active form (He et al., 2016; Jo et al., 2016), which mediates the maturation and secretion of IL-1β and IL-18, and the cleavage of GSDMD, a key executor of pyroptosis (Kovacs and Miao, 2017; Shi et al., 2017; Kang et al., 2018). The existence of pyroptosis in cardiomyocytes induced by sepsis was proved by several studies (Li et al., 2021; Xiong et al., 2022), however its regulatory mechanism remains unclear and needs further research.

Mitochondrial homeostasis is essential for myocardial survival. Mitochondrial fatty acid β-oxidation is the major pathway for fatty acid degradation and energy supply of cardiomyocytes, and its disturbance leads to energy shortage and mitochondrial instability (Houten et al., 2016; Panov et al., 2022). It has been reported that mitochondrial instability is closely associated with NLRP3 inflammasome activation (Zhou et al., 2011; Wang et al., 2020). However, whether fatty acid β-oxidation disorder is associated with NLRP3 inflammasome activation and cardiomyocyte pyroptosis remains unknown. Hydroxyacyl-CoA dehydrogenase trifunctional multienzyme complex α subunit (HADHA), known as the α-subunit of mitochondrial trifunctional enzyme complex, is a key protein for fatty acid β-oxidation (Liu et al., 2020), the enzymatic activity of which can be regulated by acetylation at Lysine 303 (Chi et al., 2020). Loss of HADHA activity or mutations in its encoding gene can lead to cardiomyopathy and other disorders (Miklas et al., 2019).

Mitochondrial aldehyde dehydrogenase (ALDH2) is well-known for its role in maintaining mitochondrial homeostasis through aldehyde scavenging and antioxidant effects (Chen et al., 2014). *Aldh2* rs671 genetic mutation is an independent risk factor for cardiovascular diseases (Pang et al., 2019; Bartoli-Leonard et al., 2020), and about 30%–50% of East Asian individuals carry this mutation, causing a 90% loss of its enzyme activity (Gross et al., 2015). However, as a protein mainly located in mitochondria, it is still unclear whether ALDH2 affects fatty acid β-oxidation and plays a role in myocardial pyroptosis.

This study will demonstrate the role of ALDH2 as a therapeutic target for myocardial pyroptosis, and elucidate novel mechanisms focusing on the mitochondrion-NLRP3 inflammasome interaction.

## Materials and methods

### Animals and treatment

Six to 8 weeks old C57BL/6J mice, *Aldh2* knockout mice (*Aldh2* ^−/−^) were used. Only male mice were chosen for experiment to avoid sex hormone interference. Mice were housed in an appropriate environment (23.0°C ± 2.0°C, 45%–50% humidity) with a 12/12-light/dark cycle, and they had access to food and water *ad libitum* until experimentation. They were randomly divided into different groups. Model was established by administrating 15 mg/kg of Lipopolysaccharide (LPS, Sigma) intraperitoneally (i.p.) 12 h before sacrifice. Alda-1 (20 mg/kg i.p., Sigma-Aldrich) or necrosulfonamide (NSA, 20 mg/kg i.p., Abcam) was administered half an hour before LPS injection. An equal amount of pathogen-free normal saline (NS) was used as control.

### Cell culture and treatment

The rat cardiomyocyte cell line (H9C2) was cultured in a high-sugar medium (DMEM, Gibco) containing 1% penicillin/streptomycin and 10% fetal bovine serum (FBS, Gibco), and were exposed to 95% O_2_ and 5% CO_2_ at 37°C. After starving with serum-free medium overnight and replacing with complete medium, H9C2 cells were stimulated by LPS (2 μg/mL, 24 h) and ATP (40 μmol/L, 45 min) with or without Alda-1 (20 μmol/L, 30 min before LPS challenge). And H9C2 cells were stimulated by 4-HNE (40 μmol/L, 12 h) with or without Alda-1 (20 μmol/L, 30 min before stimulation).

### Echocardiography

Septic mice were anesthetized (inhaled 2% isoflurane) for echocardiography, which was performed by a Vevo770 imaging system. Two-dimensional and M-mode images of the heart were collected. Cardiac function was measured in at least five repetitive cardiac cycles. The subsequent procedure was to calculate the left ventricular ejection fraction (LVEF%), fraction shortening (FS%), and heart rate (HR).

### Cytokine measurement

Mouse cardiac IL-1β, IL-6 levels were measured using R&D ELISA kits, TNF-α was measured *via* eBioscience ELISA kit. The levels of mice IL-18 were measured using RayBiotech ELISA kit. The levels of mice LDH were measured with a biochemical analyzer (Mindray, Shenzhen, China).

### ALDH2 activity

The mitochondria were isolated from myocardial tissue using the issue mitochondria isolation kit (Beyotime) according to the manufacturer’s instructions. Mitochondrial protein concentration was detected. Then samples were incubated with sodium pyrophosphate, NAD^+^, and propionaldehyde for 10 min. NAD^+^ was reduced to NADH, which was used to determine ALDH2 activity. Production of NADH was determined by spectrophotometric absorbance at 340 nm. ALDH2 activity was expressed as nmol NADH/min per mg protein (Wang et al., 2011).

### Murine sepsis score

Murine sepsis score was used to evaluate the severity of sepsis (Shrum et al., 2014; Sulzbacher et al., 2020). The score includes seven aspects, namely, appearance, level of consciousness, activity, response to stimulus, eyes, respiration rate, and respiration quality; each part is divided into 0–4 levels, total score ranges from 0 to 28.

### Morphological imaging of pyroptosis

To observe the morphology of cardiomyocytes, the cells were seeded in confocal dish, which were stimulated by LPS (2 μg/mL, 24 h) and ATP (40 μmol/L, 6 h) with or without Alda-1 (20 μmol/L, 30 min before LPS challenge). PI (1 μg/mL, 10 min) was added to the culture medium to evaluate cell membrane integrity. Static bright-field and fluorescent images of pyroptotic cells were captured using a confocal microscope (Leica, Wetzlar, Germany). All imaging data are representative of at least three randomly selected fields. The images were processed using ImageJ software. Results were expressed as the number of PI-positive cells/total cells × 100%.

### Dihydroethidium fluorescence staining

Dihydroethidium fluorescence staining was used to assess the production of ROS. The samples were incubated with dihydroethidium solution (5 μmol/L; Beyotime Biotechnology) in a light-protected humidified chamber at 37°C for 30 min. The images were observed under a fluorescence microscope (Olympus, BX43, Tokyo, Japan).

### Transferase-mediated dUTP Nick-End labeling (TUNEL) staining

An ApopTag^®^ *In Situ* Apoptosis Detection Kits (Millipore) was used for TUNEL staining. The sections were observed and photographed under a fluorescence microscope (Olympus, BX43, Tokyo, Japan). Results were expressed as the number of TUNEL-positive cells/total cells × 100%.

### Immunohistochemical staining

Heart tissue paraffin sections were dewaxed in xylene and dehydrated in a graded series of ethanol. Following this, the sections were incubated with 3% H_2_O_2_ and blocked with 5% BSA. For immunohistochemical analyses, the sections were probed with primary antibody against caspase-1 (1:100, CST) and then incubated with DAB. Finally, the sections were observed and photographed under microscope.

### Immunofluorescence staining and NLRP3/ASC speck formation

In short, the samples were infiltrated with 0.1% Triton X-100 and blocked with 5% goat serum, then incubated with primary antibody at 4°C overnight. The secondary antibody was incubated for 2 h, then incubated with DAPI to label the nucleus. The NLRP3/ASC speck formation is a sign of NLRP3 inflammasome activation by double staining for NLRP3 (ABclonal 1:200) and ASC (Abcam 1:200). The images were observed under fluorescence microscope or confocal microscope.

### Nuclear, cytoplasmic and mitochondrial protein extraction

Cytoplasmic and nuclear proteins were extracted using NE-PER Nuclear and Cytoplasmic Extraction Reagents (Thermo) according to the manufacturer’s instructions. In brief, H9C2 cells were harvested with trypsin-EDTA and lysed in CER. After centrifuging at 15,000 rpm for 5 min, the supernatant was collected as a cytosolic fraction. The remaining pellet was suspended in NER, and the supernatant was collected as nuclear fraction.

Mitochondria were isolated by cell mitochondrial isolation kit (Beyotime). In brief, after lysing and centrifuging at first time, the cell-debris pellet in the collection tube was used to extract nuclear protein, the supernatant was transferred to a new microcentrifuge tube and centrifuged at 16,000 g for 10 min, the remaining pellet was suspended with lysis buffer as mitochondrial protein. Nuclear protein was extracted using NE-PER Nuclear and Cytoplasmic Extraction Reagents (Thermo).

### Flow cytometry

After treatment, H9C2 cells were stained at RT for 60 min with FAM-FLICA working solution (ImmunoChemistry Technologies) and mixed every time 15 min, followed by the addition PI staining for 5 min at RT. After being washed three times with wash buffer, cells were trypsinized to suspend. The FAM-FLICA/PI double-positive ratio was analyzed with Cytoflex cytometry and related analyzer software (Beckman, California, United States).

### Small-interfering RNA transfection

H9C2 cells were plated in 6-well plates and transfected with 100 nM siRNA using Lipofectamine RNAiMAX (Thermo Fisher Scientific) according to the manufacturer’s instructions. The siRNA sequences for rat *Aldh2* (5′-GUG​GAU​GAG​ACU​CAG​UUU​ATT-3′), rat *Hdac3* (5′-GGG​AAU​GUG​UUU​GAA​UAU​GUT​T-3′) and rat *Hadha* (5′-GUG​UAG​AAC​UGC​UGA​AAC​UTT-3′); and negative control were synthesized by Genepharma (Shanghai, China).

### Mitochondrial respiration function

Mitochondrial respiratory function was detected with the Seahorse XF Cell Mito Stress Test Kit (Agilent Technologies, Santa Clara, California) using Seahorse XFe96 analyzer. Briefly, 1*10^6^ cells were seeded in a cell culture microplate. After the treatment, cells were equilibrated with XF assay media. Sensor cartridge was hydrated in Calibrant at 37°C in a non-CO_2_ incubator overnight. Then, the mitochondrial respiratory function was analyzed by XFe96 analyzer with compounds.

### Transfection of mtDNA

Mitochondria were isolated by tissue or cell mitochondrial isolation kit (Beyotime), according to the manufacturer’s instructions. mtDNA was isolated from the mitochondrial pellet with a DNeasy blood and tissue kit (Qiagen). The mtDNA was incubated with 100 mM hydrogen peroxide for 50 min at 37°C to generate oxidized mtDNA. H9C2 cells were transfected isolated mtDNA or oxidized mtDNA (2 mg/mL, 6 h) *via* Attractene (Qiagen) according to the manufacturer’s instructions.

### Measurement of oxidative mtDNA

Mitochondria DNA was extracted from H9C2 cells by Allprep tissue/cell RNA-DNA extraction kit (Aidlab Biotech) according to the manufacturer’s instructions. The oxidative mtDNA was measured with 8-OHdG quantification kit (Cell Biolabs) which quantified the levels of 8-OHdG (the marker of oxidized DNA), as the manufacturer’s instructions.

### Western blot and co-immunoprecipitation

Protein samples were separated by 8%–12% SDS-PAGE and transferred to nitrocellulose membranes (Millipore). After blocking with 5% milk in TBST for 1h, the membranes were incubated overnight at 4°C with primary antibodies. Subsequently, membranes were washed and incubated with secondary antibodies (1:10,000) and detected using the chemiluminescence method. The intensity of the bands was quantified by ImageJ software. Antibodies included anti-NLRP3 antibody (1:1,000, CST), anti-GSDMD antibody (1:1,000, Abcam), anti-N-GSDMD antibody (1:1,000, Abcam), anti-Capspase-1 p20 antibody (1:1,000, AdipoGen), anti-4-HNE antibody (1:1,000, Abcam), anti-HDAC3 antibody (1:1,000, Proteintech), anti-HADHA antibody (1:1,000, Abcam), anti-Ace-lys antibody (1:1,000, Abcam), anti-H3 antibody (1:1,000, Proteintech), anti-COX4 antibody (1:1,000, Abcam), anti-PCNA antibody (1:1,000, Abcam). Anti-GAPDH (1:5,000, Invitrogen) was used as an internal control.

For acetylation-immunoprecipitation, cells were lysed with immunoprecipitation buffer [supplemented with TSA (10 mM)] and sonicated. The samples were immunoprecipitated with protein A/G beads (Sigma) overnight at 4°C, washed three times in lysis buffer, resolved by loading buffer, and analyzed by Western blotting.

### Reverse transcription quantitative polymerase chain reaction

The total RNA was extracted from cardiac tissue with TRIzol (Invitrogen), according to the manufacturer’s instructions. Then, 2 μg of total RNA was reverse transcribed into cDNA. The 7500 quantitative polymerase chain reaction machine (Thermo Fisher Scientific) was used for real-time polymerase chain reaction analysis. Actin was used as an internal control.

### Statistical analysis

The continuous data were presented as mean ± SEM. Group comparisons were performed by one-way analysis of variances (ANOVA) with Tukey’s *post hoc* test or Student’s t-test. Survival was presented by Kaplan-Meier curves, and the log-rank test was used for comparing survival rate between groups. *p* < 0.05 was considered statistically significant (2-tailed).

## Results

### *Aldh2* knockout aggravates septic shock-induced cardiac dysfunction and mortality

**FIGURE 1:**
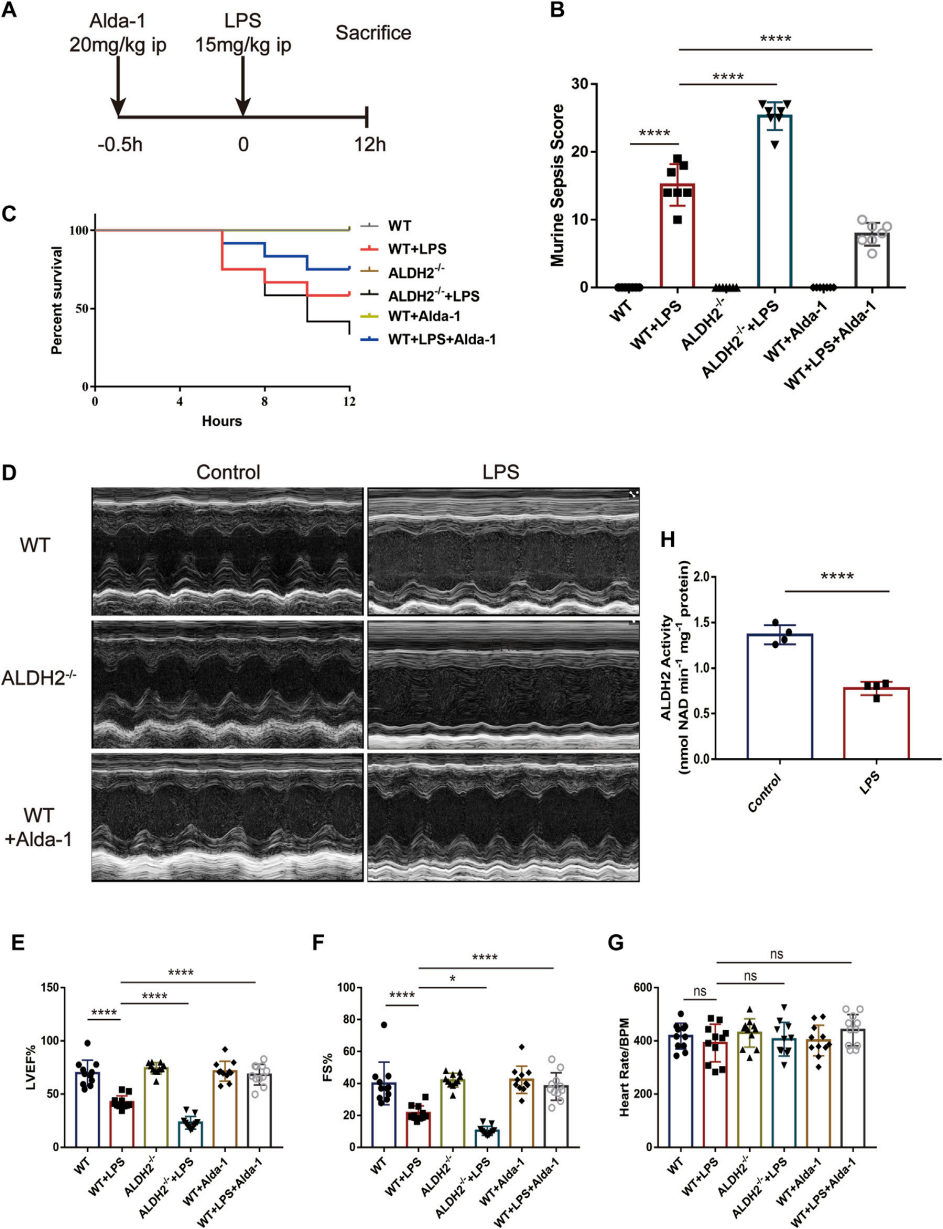
Effect of ALDH2 on LPS-induced cardiac dysfunction and mortality. **(A)** Experimental modeling. Mice were treated with or without LPS (15 mg/kg, i.p. for 12 h). Alda-1 (20 mg/kg, i.p.) was given 30min before LPS injection. **(B)** Murine Sepsis Score, *n* = 7 per group. **(C)** Survival rate was monitored up to 12 h. A Kaplan-Meier plot was used to show the survival rate of mice from each group, *n* = 12 per group. **(D–G)** Representative echocardiographic images from different groups and the quantitative analysis of echocardiography, *n* = 11 per group. **(H)** The quantitative analysis of ALDH2 enzymatic activity, *n* = 4 per group, Mean ± SEM. ****p < 0.0001; ***p < 0.001; **p < 0.01; *p < 0.05; ns = not significant.

The mice model of septic shock was established by intraperitoneal injection of LPS as shown in Figure 1A. Compared with the control group, LPS treatment induced a very high murine sepsis score, while *Aldh2* knockout (*Aldh2* ^−/−^ genotype) and Alda-1 treatment further aggravated and reduced the score relative to the LPS group, respectively (Figure 1B). To further clarify the beneficial effect of ALDH2 activation, we also studied the role of its activator Alda-1 on the short-term (12 h) survival rate of septic mice. In the septic shock group, the 12-hour survival rate was 55.5%, *Aldh2* knockout exacerbated the survival rate to 33.3%, while Alda-1 treatment improved the survival rate to 80% (Figure 1C). Results of cardiac echocardiography showed that, compared with the control group, LPS significantly reduced the left ventricular ejection fraction (LVEF%) and fraction shortening (FS%), which is consistent with the results of our previous studies (Pang et al., 2019). Although *Aldh2* ^−/−^ genotype *per se* did not affect cardiac function, *Aldh2* knockout exacerbated LPS-induced cardiac dysfunction, while Alda-1 significantly ameliorated it (Figures 1D–G). LPS was also found to significantly reduce the activity of ALDH2 in cardiac tissue of wild-type mice (Figure 1H).

### ALDH2 attenuates myocardial pyroptosis through NLRP3 inflammasome/caspase-1/GSDMD pathway in septic shock

**FIGURE 2:**
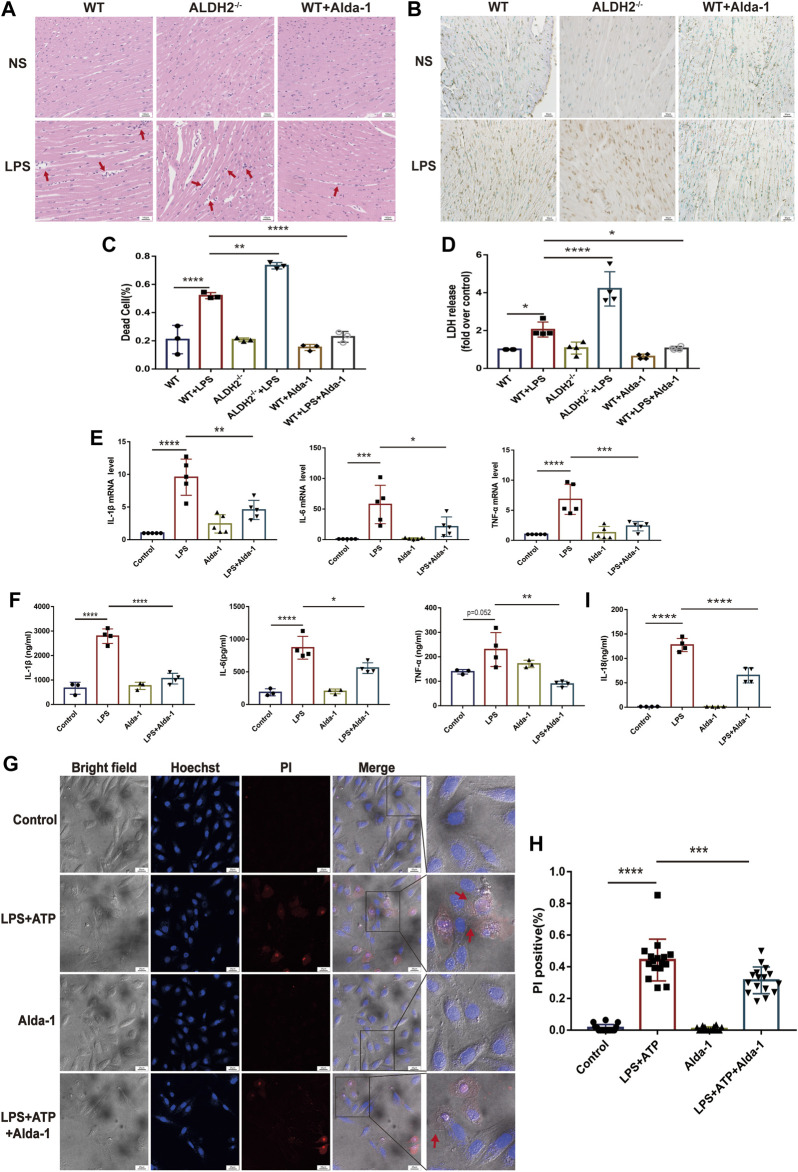
ALDH2 attenuates LPS-induced myocardial pyroptosis and inflammation in septic shock. **(A)** Representative HE staining images of cardiac tissue, red arrows indicate inflammatory infiltration, scale bar: 100 μm. **(B,C)** Representative TUNEL staining images of cardiac tissue and the quantitative analysis, scale bar: 50 μm. **(D)** The quantitative analysis of LDH release levels, *n* = 4 per group. **(E,F)** The quantitative analysis of RT-qPCR and ELISA results including IL-1β, IL-6, and TNF-α. N = 3–5 per group. **(G,H)** Representative morphological changes of pyroptosis in bright field and PI staining and the quantitative analysis of the PI positive H9C2 cells, red arrows indicate bubbling of pyroptotic cells, scale bar: 25 μm. **(I)** The quantitative analysis of IL-18 release level, *n* = 4 per group. Mean ± SEM, ****p < 0.0001, ns = not significant.

To evaluate the effect of ALDH2 on cardiac injury and the potential mechanisms, we performed staining to assess myocardial morphology and cell death. Compared with the control group, LPS challenge increased inflammatory cell infiltration and broken myocardial fibers as revealed *via* hematoxylin-eosin (HE) staining (Figure 2A), and overtly elevated the numbers of dead cardiomyocytes as showed by TUNEL staining (Figures 2B, C), which were further aggravated and effectively alleviated in *Aldh2* knockout (*Aldh2* ^−/−^) mice and mice pre-treated with Alda-1, respectively. The level of plasma LDH, a marker of organ damage, also indicated the protective effect of ALDH2 in sepsis (Figure 2D). As for the inflammatory cytokines IL-1β, IL-6, and TNF-α, the results showed that the transcription and expression levels of these cytokines were markedly elevated by LPS stimulation (except the expression of TNF-α, *p* = 0.052). Alda-1 pretreatment did significantly reduce the transcription and expression levels of these cytokines (Figures 2E, F). The plasma IL-18 level was also consistent with these results (Figure 2I). Collectively, the above results suggest that ALDH2 attenuates inflammation and cardiomyocyte death in septic shock-induced cardiac dysfunction.

**FIGURE 3:**
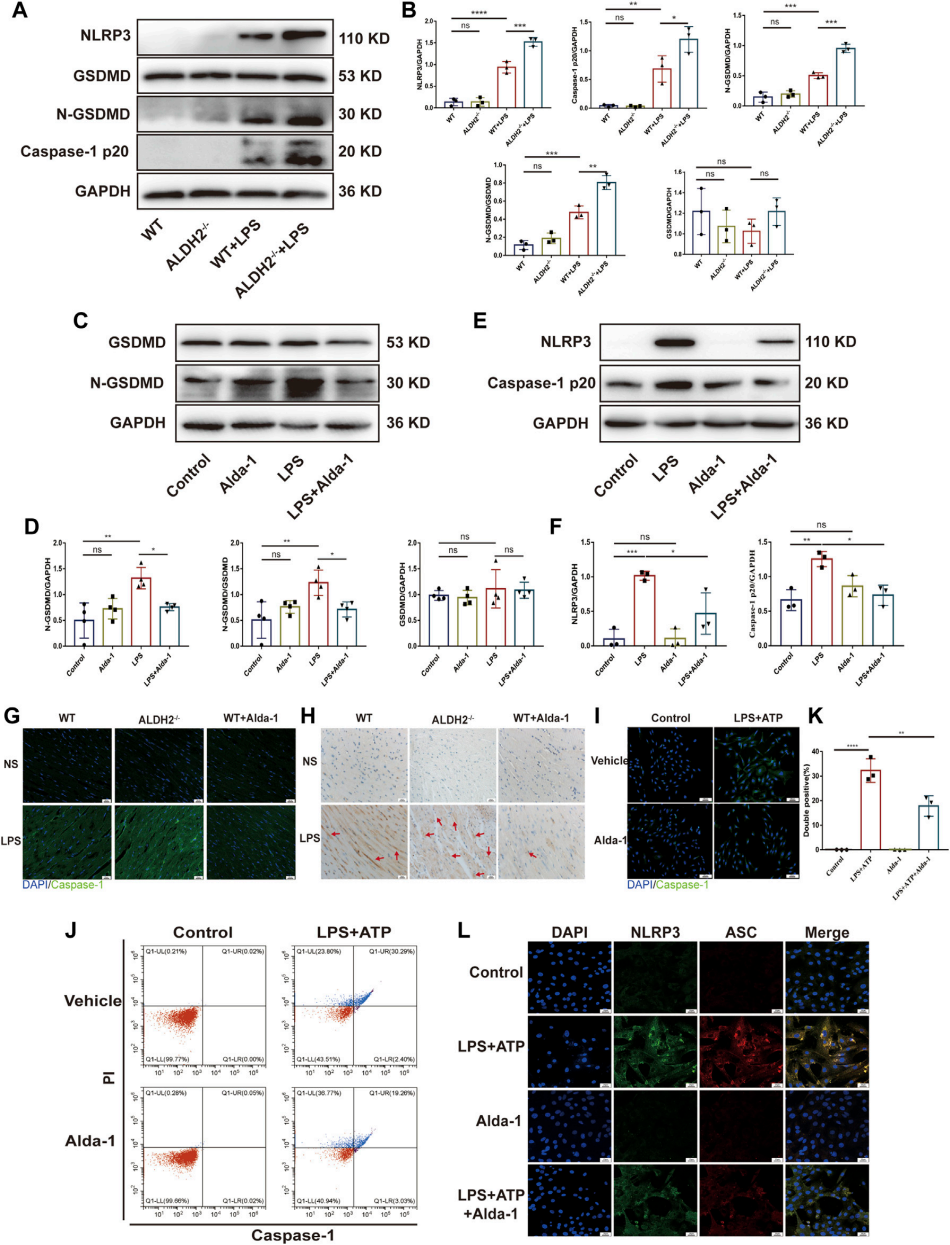
ALDH2 inhibits myocardial pyroptosis through NLRP3/Caspase-1/GSDMD signaling pathway. **(A-F)** Representative immunoblots and quantification of N-GSDMD, GSDMD, NLRP3 and caspase-1 p20 protein in LPS-stimulated *Aldh2* ^−/−^ and WT mice (GAPDH; loading control), *n* = 3-4 per group. **(G)** Representative immunofluorescence tissue images showing caspase-1 (green) and DAPI (blue), scale bar: 50 μm. **(H)** Representative immunohistochemical images showing caspase-1 (brown), scale bar: 50 μm. **(I–L)** H9C2 cells were stimulated by LPS plus ATP with or without pre-treated Alda-1. **(I)** Representative immunofluorescence images showing caspase-1 (green) and DAPI (blue), scale bar: 100 μm. **(J,K)** Representative flow cytometry graphs showing positive-caspase-1/PI double staining and the quantitative analysis of the double positive, *n* = 3 per group. **(L)** Representative immunofluorescence images showing NLRP3/ASC speck, scale bar: 25 μm. Mean ± SEM. ****p < 0.0001; ***p < 0.001; **p < 0.01; *p < 0.05; ns = not significant.

To further validate the occurrence of pyroptosis, we performed immunofluorescence and observed the typical morphological changes of pyroptosis. In response to LPS and ATP stimulation, H9C2 cells firstly underwent deformation, and then the cell membrane gradually expanded, developed bubble-like herniations, and finally the cell membrane ruptured. Alda-1 was found to alleviate these changes (Figures 2G, H). To further clarify the role of pyroptosis in septic shock-induced cardiac dysfunction, necrosulfonamide (NSA), a well-recognized chemical inhibitor of pyroptosis (Rathkey et al., 2018), was administered 30 min prior to LPS treatment to C57/BL6 male mice, and it significantly improved LPS-induced cardiac dysfunction as shown in echocardiography (Supplementary Figure S1). To further validate the role of ALDH2 in pyroptosis, we evaluated the protein expression of N-GSDMD, a key marker that mediates membrane perforation in pyroptosis. Compared to wild-type mice, LPS challenge triggered even a higher level of N-GSDMD and N-GSDMD/GSDMD ratio in *Aldh2* ^−/−^ mice (Figures 3A, B). On the contrary, pretreatment with Alda-1 significantly reduced the LPS-induced upregulated levels of N-GSDMD and the N-GSDMD/GSDMD ratio (Figures 3C, D). These findings illustrated that ALDH2 protects against septic shock-induced cardiac dysfunction through inhibiting cardiomyocyte pyroptosis.

NLRP3 inflammasome/Caspase-1 signaling pathway is the canonical pathway of pyroptosis (Jo et al., 2016; Gaul et al., 2021). We investigated the protein levels of NLRP3 and the activated Caspase-1 form Caspase-1 p20, a sign of NLRP3 inflammasome activation. Compared to wild-type mice, LPS challenge triggered even a higher level of NLRP3 and Caspase-1 p20 in *Aldh2* ^−/−^ mice (Figures 3A, B). On the contrary, pretreatment with Alda-1 significantly reduced LPS-induced upregulated levels of NLRP3 and Caspase-1 p20 (Figures 3E, F). Both immunohistochemistry staining and immunofluorescence staining showed increased expression of Caspase-1 in LPS-challenged cardiac tissue, which was further enhanced in *Aldh2* ^−/−^ mice, while Alda-1 pretreatment significantly reversed it (Figures 3G, H). The results of flow cytometry and Caspase-1 immunofluorescence staining were consistent in H9C2 cells, which showed increased the number of double positive ratio and increased expression of Caspase-1 in LPS and ATP group compared with the control group, while the changes were attenuated by Alda-1 pretreatment (Figures 3I–K). We also examined NLRP3/ASC speck formation, another sign of NLRP3 inflammasome activation (Qiao et al., 2021). We observed an increase in the number of cells with NLRP3/ASC specks induced by ATP in LPS-primed cells compared with Alda-1 pretreatment cells (Figure 3L). These results demonstrated that activated ALDH2 attenuated NLRP3 inflammasome activation. Collectively, these findings demonstrated that ALDH2 may protect against septic shock-induced cardiac dysfunction through inhibiting cardiomyocyte pyroptosis mediated by NLRP3 inflammasome/Caspase-1/N-GSDMD pathway.

### ALDH2 inhibited the activation of NLRP3 inflammasome through reducing mtDNA and ox-mtDNA

**FIGURE 4:**
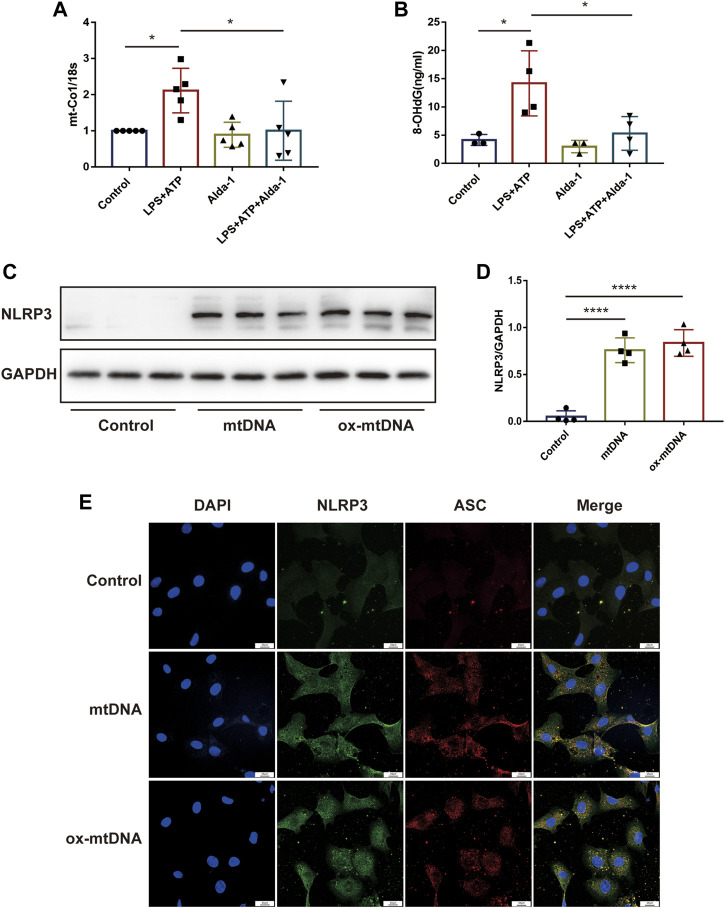
MtDNA and ox-mtDNA promotes NLRP3 expression and NLRP3 inflammasome activation. **(A,B)** The levels of total mtDNA or 8-OHdG (ox-mtDNA) in LPS plus ATP-stimulated or control H9C2 cells pre-incubated with or without Alda-1, *n* = 3-5 per group. **(C,D)** Representative immunoblots and the relative quantification analysis of NLRP3 with or without mtDNA or ox-mtDNA transfection, *n* = 4 per group. **(E)** Representative immunofluorescence images showing NLRP3/ASC speck, scale bar: 25 μm. Mean ± SEM. ****p < 0.0001; ***p < 0.001; *p < 0.05; ns = not significant.

To explore the potential role of ALDH2 in regulating the mitochondrion-NLRP3 inflammasome pathway, we performed *in vitro* studies focusing on the mitochondrial DNA release. Compared with the control group, LPS in addition to ATP significantly increased the cellular level of mtDNA as well as ox-mtDNA, while ALDH2 activation remarkably reduced LPS-induced mtDNA and ox-mtDNA release (Figures 4A, B). To further verify the roles of mtDNA and ox-mtDNA in NLRP3 inflammasome activation, mtDNA or ox-mtDNA was transfected to the H9C2 cells *in vitro*. Western blot and fluorescence staining results showed that both mtDNA and ox-mtDNA transfection directly elevated the expression of NLRP3 and NLRP3/ASC speck formation (Figures 4C–E). Collectively, these data suggest that mitochondrial damage plays an important role in mediating NLRP3 inflammasome activation, which may be through the release of mtDNA and ox-mtDNA.

**FIGURE 5:**
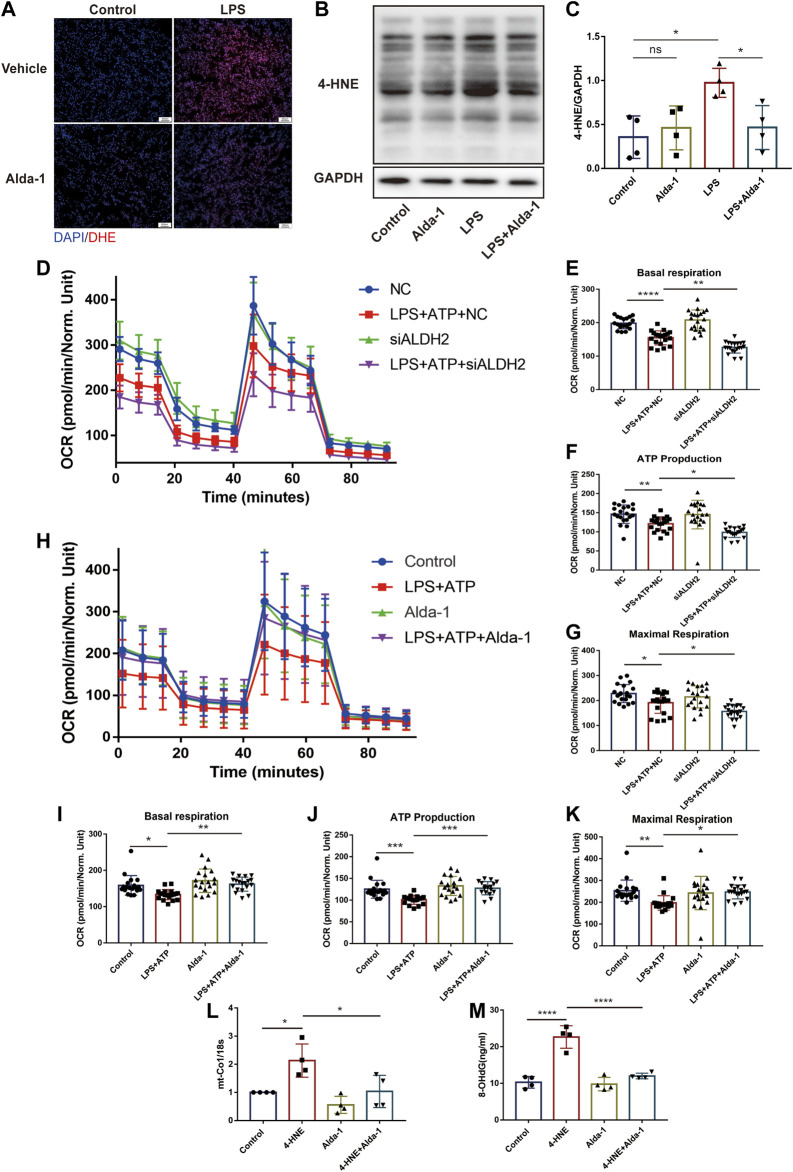
ALDH2 suppresses myocardial toxic aldehyde accumulation, oxidative stress, and improves mitochondrial respiratory function. **(A)** Representative dihydroethidium (DHE) staining images in cardiac tissue, scale bar: 200 μm. **(B,C)** Representative immunoblots and quantification of 4-HNE-protein adducts in cardiac tissue, the LPS stimulated mice were pre-treated with or without Alda-1, *n* = 4 per group. **(D–K)** Mitochondrial respiration measurements of OCR in H9C2 cells treated with *Aldh2* siRNA or negative control (NC) or Alda-1, quantification of basal respiration, ATP production, and maximal respiration. **(L,M)** The levels of total mtDNA or ox-mtDNA in 4-HNE-stimulated or control H9C2 cells pre-incubated with or without Alda-1, *n* = 4 per group. Mean ± SEM. ****p < 0.0001; ***p < 0.001; **p < 0.01; *p < 0.05; ns = not significant.

Results of dihydroethidium (DHE) staining showed that LPS triggered excessive oxidative stress in murine cardiac tissue, an effect that was significantly reduced by ALDH2 (Figure 5A). Toxic aldehyde 4-hydroxynonenal (4-HNE) as a product of oxidative stress interacting with lipids, is a marker of oxidative stress. LPS challenge significantly increased the accumulation of protein adducts of 4-HNE, which was significantly reduced by ALDH2 activation (Figures 5B, C). Consistent with those observations, mitochondrial respiratory function reflected by the reduced oxygen consumption rate (OCR) was altered by LPS and ATP treatment, and was aggravated by *Aldh2* knockdown in H9C2 cells (Figures 5D–G), while it was reversed by Alda-1 pretreatment (Figures 5H–K). In addition, we found that ROS scavenger NAC significantly improved mitochondrial damage caused by LPS and ATP treatment (Supplementary Figure S2A). To verify the effect of ALDH2 on mtDNA/ox-mtDNA release, H9C2 cells were stimulated by 4-HNE with or without Alda-1. Compared with the control group, 4-HNE significantly increased mtDNA and ox-mtDNA release, while ALDH2 reduced it, suggesting that sepsis triggered mtDNA and ox-mtDNA release through oxidative stress (Figures 5L, M).

### ALDH2 elevated acetylation level of HADHA through suppressing the translocation of HDAC3 from nuclei to mitochondria

**FIGURE 6:**
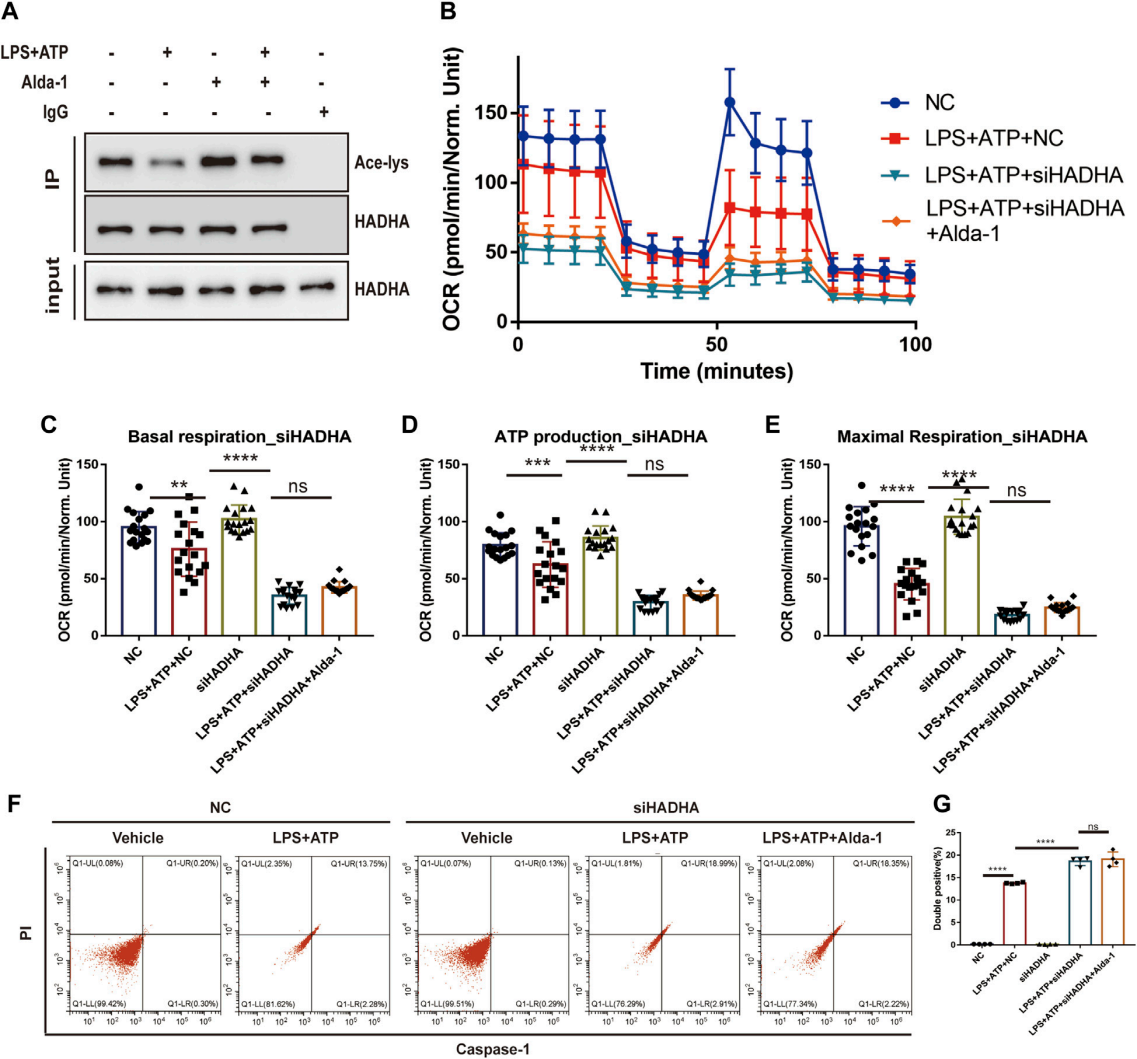
HADHA deacetylation is responsible for mitochondrial damage and cardiomyocytes pyroptosis, and ALDH2 suppresses HADHA deacetylation. **(A)** Representative immunoblots of HADHA acetylation levels in LPS plus ATP-stimulated or control H9C2 cells with or without pre-treated Alda-1, *n* = 3 per group. **(B–E)** Mitochondrial respiration measurements of OCR in *Hadha* silenced or negative control H9C2 cells with or without LPS plus ATP stimulation, quantification of basal respiration, ATP production, and maximal respiration. **(F,G)** Representative flow cytometry graphs showing positive-caspase-1/PI double staining in *Hadha* silenced or negative control H9C2 cells with or without LPS plus ATP stimulation and quantitative analysis of the double positive, *n* = 4 per group. Mean ± SEM. ****p < 0.0001; ***p < 0.001; *p < 0.05; ns = not significant.

To further elaborate the mechanism by which ALDH2 regulates mitochondrial homeostasis, we focused on mitochondrial membrane proteins. It was found that although HADHA protein levels were unchanged, sepsis significantly reduced HADHA acetylation levels compared with controls, which was reversed by ALDH2 (Figure 6A). To further determine the role of HADHA in mitochondrial respiratory dysfunction, we silenced *Hadha* in H9C2 cells with siRNAs and found that *Hadha* knockdown impaired ALDH2-induced improvement of OCR (Figures 6B–E). Moreover*, Hadha* knockdown effectively impaired ALDH2-induced improvement of pyroptosis after LPS and ATP stimulation (Figures 6F, G). These results indicate that deacetylation of HADHA is responsible for mitochondrial damage and cardiomyocytes pyroptosis.

**FIGURE 7:**
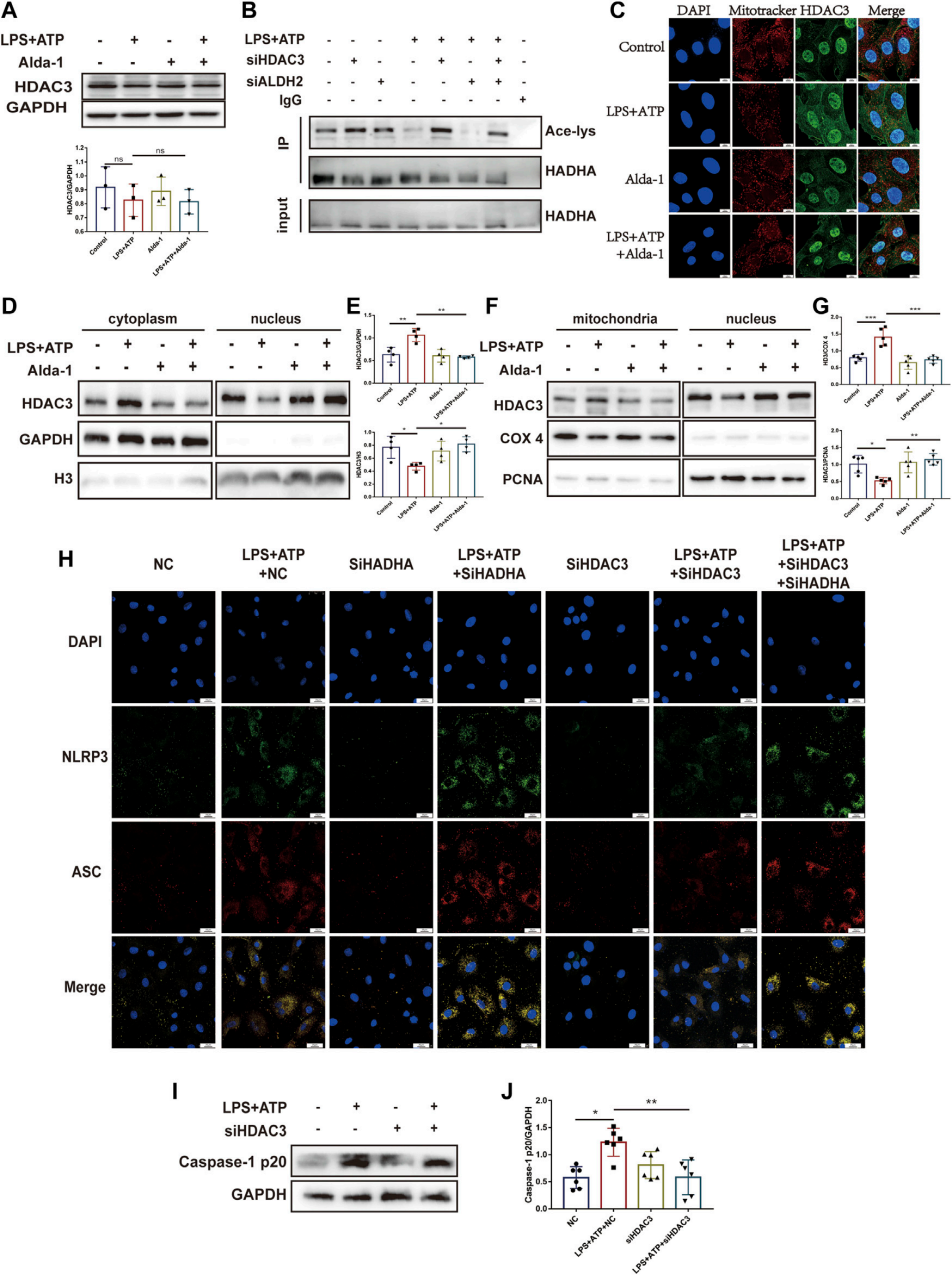
ALDH2 suppresses the translocation of HDAC3 from nucleus to mitochondria. **(A)** Total levels of HDAC3 and the quantitative analysis, *n* = 3 per group. **(B)** Representative immunoblots of HADHA acetylation levels in *Hdac3* silenced or *Aldh2* silenced or negative control H9C2 cells with or without LPS plus ATP stimulation, *n* = 3 per group. **(C)** Representative confocal images showing HDAC3 (green), Mito Tracker (red) and DAPI (blue), scale bar: 10 μm. **(D,E)** Total levels of HDAC3, and the cytoplasmic and nuclear levels of HDAC3 proteins, and the quantitative analysis. Histone 3 and GAPDH were used as loading control of nuclear and cytoplasmic fractions, respectively, *n* = 3-4 per group. **(F,G)** The level of HDAC in mitochondrial and nuclei and the quantitative analysis. PCNA and COX4 were used as loading control of nuclear and mitochondrial fractions, respectively, *n* = 5 per group. **(H)** Representative immunofluorescence images showing NLRP3/ASC speck in *Hdac3* silenced or *Hadha* silenced or negative control H9C2 cells with or without LPS plus ATP stimulation, scale bar: 25 μm. **(I,J)** Representative immunoblots and the quantification of Caspase-1 p20 protein in H9C2 cells (GAPDH; loading control), *n* = 6, Mean ± SEM. ****p < 0.0001; ***p < 0.001; *p < 0.05; ns = not significant.

Histone deacetylase 3 (HDAC3) is an important acetylase enzyme (Chen et al., 2012; Nguyen et al., 2020). LPS and ATP treatment with or without Alda-1 did not affect the total protein level of HDAC3 (Figure 7A). Interestingly, we found that *Hdac3* knockdown effectively increased HADHA acetylation level (Figure 7B). So we evaluated whether the location of HADC3 altered. Interestingly, after LPS plus ATP stimulation, HDAC3 obviously translocated from nuclei to mitochondria compared to the control group, and ALDH2 inhibited the translocation of HDAC3 as evidenced by WB and fluorescence colocalization staining (Figures 7C–G). *Hadha* knockdown and *Hdac3* knockdown obviously increased and decreased the number of cells with NLRP3/ASC speck triggered by ATP in LPS-primed cells, respectively. When *Hadha* and *Hdac3* were knocked down at the same time, *Hadha* knockdown effectively impaired the beneficial effect of *Hdac3* knockdown for inhibiting NLRP3 inflammasome activation after LPS and ATP stimulation (Figure 7H). *Hdac3* knockdown also effectively inhibited the protein level of Caspase-1 p20 (Figures 7I, J). To verify whether ALDH2 affects the acetylation level of HADHA by regulating HDAC3, we interfered with *Hdac3* and *Aldh2* at the same time. It was found that *Aldh2* knockdown significantly aggravated the decreased acetylation level of HADHA induced by LPS and ATP, however the adverse consequences of which was eliminated by *Hdac3* knockdown (Figure 7B). It was validated that ALDH2 affected HADHA acetylation *via* regulating HDAC3.

**FIGURE 8:**
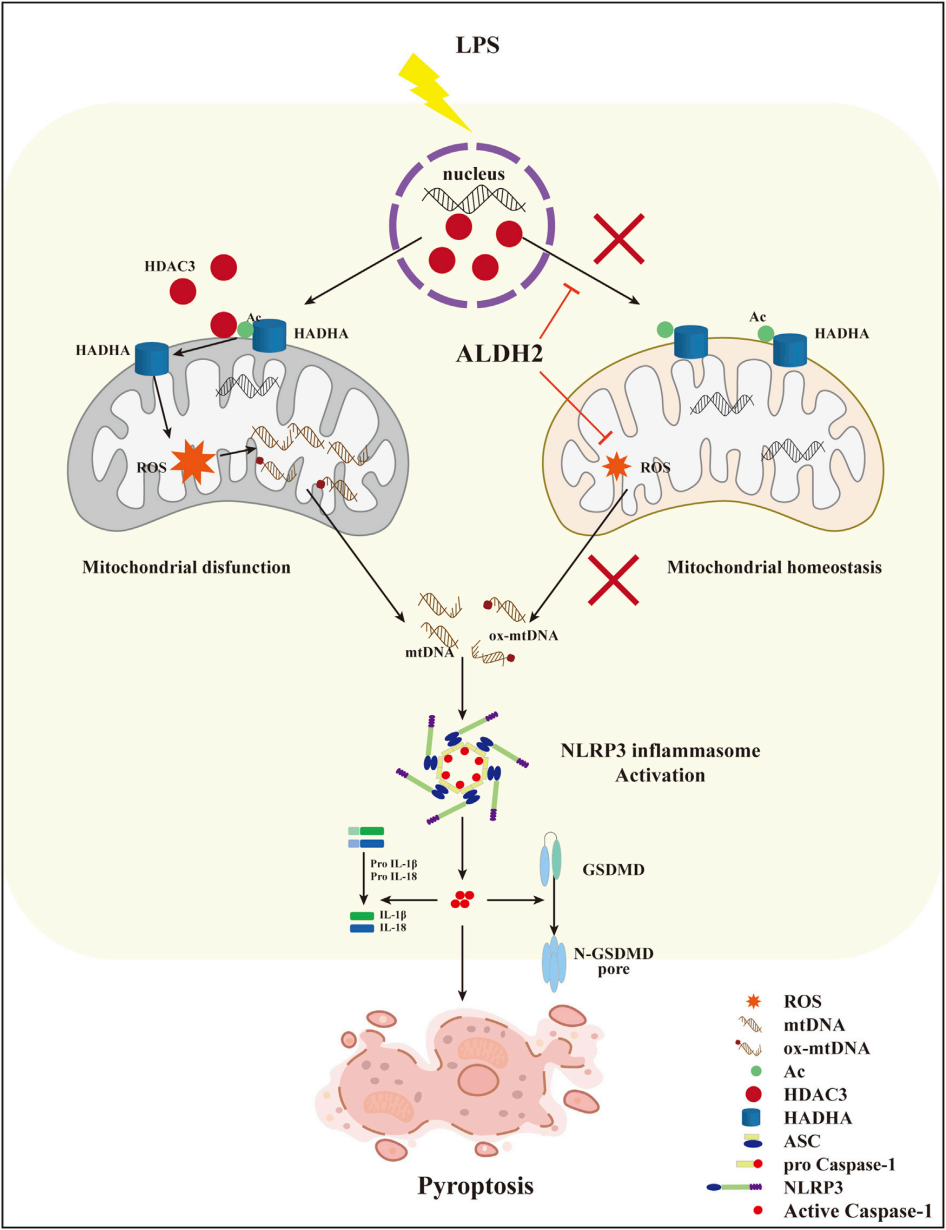
A diagram showing ALDH2 as a therapeutic target to protect against septic shock-induced myocardial pyroptosis.

Collectively, these findings demonstrated that ALDH2 may protect against myocardial pyroptosis through mitochondrial HDAC3/HADHA-NLRP3 inflammasome pathway in septic shock (Figure 8).

## Discussion

This study illustrated that septic shock might trigger NLRP3/Caspase-1/GSDMD-dependent myocardial pyroptosis due to activating the mitochondrion-NLRP3 inflammasome pathway *via* the release of mtDNA and ox-mtDNA. LPS promoted the translocation of HDAC3 from the nucleus to the mitochondria and thereby increased the deacetylation of the mitochondrial fatty acid β-oxidation enzyme HADHA, disturbing mitochondrial homeostasis and leading to overwhelming mitochondrial oxidative stress, which was supposed to be responsible for the increase of mtDNA and ox-mtDNA fragments. We also found that *Aldh2* knockout and ALDH2 activation significantly aggravated and reduced myocardial pyroptosis, respectively. Alda-1, an agonist of ALDH2, remarkably reduced pyroptosis and rescued septic shock-induced cardiac dysfunction, possibly through clearing toxic aldehydes and inhibiting the translocation of HDAC3 to protecting ace-HADHA, decreasing NLRP3 inflammasome activation, and maintaining mitochondrial homeostasis. This study clarified novel mechanisms regulating myocardial pyroptosis and elucidated the underlying mechanisms of ALDH2 as a therapeutic target in myocardial pyroptosis.

In this study, we established a mouse mode of sepsis by administering LPS intraperitoneally with a relatively high dose of LPS (15 mg/kg) and successfully activated pyroptosis, indicating that myocardial pyroptosis may be less likely to be activated than apoptosis (Jiang et al., 2021). Based on the murine sepsis score and echocardiography findings, this model simulated septic shock. Cardiac dysfunction depends largely on cell death and inflammation (Mann, 2015; Zhang et al., 2017; Adamo et al., 2020). Pyroptosis is characterized by excessive inflammation and amplification of cellular injury (Bergsbaken et al., 2009), which can not only cause the death of cardiomyocytes, but also triggers a vicious circle, aggravating the inflammatory milieus and myocardial damage. Consistent with other studies, we found that inhibition of pyroptosis significantly improved the survival rate and cardiac function in sepsis. Caspase-1-dependent signaling is considered as the canonical pathway of pyroptosis (Swanson et al., 2019; Xue et al., 2019), and Caspase-11 mediates a non-canonical pathway of pyroptosis (Man and Kanneganti, 2015). Caspase family members such as Caspase-3/6/8 have recently been found to participate in pyroptosis (Fritsch et al., 2019; Zheng et al., 2020). Our study confirmed that septic shock induced myocardial pyroptosis through NLRP3 inflammasome/Caspase-1/GSDMD pathway, and inhibition of pyroptosis was found to alleviate septic shock-induced cardiac dysfunction.

The interaction between mitochondria and inflammasome, as well as its effects on diseases, have always been the focus of research (Zhong et al., 2016; Huang et al., 2020). Previous study showed that newly-synthesized ox-mtDNA led to NLRP3 inflammasome activation in macrophages (Zhong et al., 2018), while this study suggested that both oxidized- and non-oxidized mitochondrial DNA released from damaged mitochondria enhanced the expression and activation of NLRP3 in cardiomyocytes, which might be critical factors in mitochondria-NLRP3 inflammasome pathway. Oxidative stress is known to promote the formation of damaged mtDNA fragments and ox-mtDNA. We found that LPS promoted the deacetylation of HADHA, whereas HADHA could not properly catalyze the fatty acid β-oxidation process, resulting in insufficient energy supply as well as oxidative stress and impaired mitochondrial respiratory function due to the accumulation of long-chain saturated fatty acid acylcarnitine and triglyceride. When the *Hadha* gene was interfered by siRNA, similar phenomenon was observed.

The present study demonstrated for the first time the protective effect of ALDH2 on myocardial pyroptosis induced by sepsis. In this study, we also explored whether ALDH2 protected mitochondria through pathways other than aldehyde clearance and found that ALDH2 significantly rescued mitochondrial inner membrane protein HADHA from deacetylation. When the *Hadha* gene was interfered with, the beneficial effect of Alda-1 no longer exists. An increasing body of evidence has shown that acetylation of mitochondrial proteins is important for the regulation of mitochondrial function (Dittenhafer-Reed et al., 2015; Carrico et al., 2018; Deng et al., 2021). HDAC3 is widely known as an epigenetic regulator which inhibits the dissociation of DNA from histone octamer and compacts and curls the chromatin to block gene transcription. HDAC3 can shuttle between nucleus and cytoplasm and play different functions according to its location (Jiang et al., 2022). It was reported that ALDH2 could translocate to the nucleus and bind with HDAC3 to inhibit the expression of the lysosomal proton pump protein ATP6V0E2, resulting in impaired lysosomal function (Zhong et al., 2019). In this study, we found that ALDH2 remarkably inhibited the cytoplasmic shuttle of HDAC3 induced by sepsis, and it might be related to the binding of the two proteins. This study is the first to demonstrate that ALDH2 may inhibit sepsis-induced translocation of HDAC3 from nucleus to mitochondria.

Besides NLRP3, a variety of inflammasome-forming NLRs have been identified (including NLRP1/2/6/7/12, NLRB and NLRC4/5), which typically contain an evolutionarily conserved tripartite structure, consisting of a N-terminal effector domain, a central nucleotide-binding domain (NBD/NACHT) and a C-terminal autoregulatory LLR domain (Broz and Dixit, 2016; Olsen et al., 2022). However, the assembly of each is determined by unique pattern recognition receptors (PRRs) in response to pathogen-associated molecular patterns (PAMPs) or endogenous danger signals in the cytosol of the host cell (Rathinam and Fitzgerald, 2016; Olsen et al., 2022). A review published in 2021 has summarized the various activators of different inflammasomes, as shown in the figure below (Carriere et al., 2021). In our present study, we focused on the effect of ALDH2 on NLRP3 inflammasome through HDAC3/HADHA/ROS/mtDNA axis. It can be figured out that in addition to NLRP3, AMI2 inflammasome can be triggered by cytosolic DNA and may be a potential target of ALDH2. Moreover, further literature search showed that ROS production could activate NLRP1 (Xu et al., 2019; Fenini et al., 2020) and inhibits NLRP6 (Li et al., 2022). Therefore, NLRP1/6 and AIM2 are all potential downstream targets of ALDH2 in addition to NLRP3. However, whether other inflammasome-forming NLRs are possible targets of ALDH2 still need further investigation.

The dose of LPS used in our previous study was 4 mg/kg for 6 h to simulate cardiac dysfunction induced by mild sepsis (Pang et al., 2019). In this study, severe sepsis, septic shock is discussed. Mechanistically, in a severe state, cardiac function declines rapidly, ER stress and autophagy can no longer self-compensate and maintain balance, thereby led to cardiomyocyte death and impaired cardiac function. Interventions that directly target cell death during the severe shock phase may be the focus of rescue. Therefore, inflammatory programmed cell death, pyroptosis, is the key process of myocardial injury in septic shock, which is the focus of our research.

However, some limitations of our study should be considered. First, whether ALDH2 plays a role in the non-canonical pathway of pyroptosis needs further study. Second, the procedure of the release of mtDNA or ox-mtDNA from mitochondria into cytoplasm has not been investigated because of technical challenges. Third, the mechanism by which ALDH2 regulates HDAC3 has not been fully studied in this study, which will be illuminated in further research.

In conclusion, this study suggests that ALDH2 may protect against septic shock-induced myocardial pyroptosis by inhibiting the mitochondrion-inflammasome pathway through clearing aldehydes and blocking the translocation of nuclear HDAC3 into mitochondria and the consequent HADHA deacetylation. This study elucidates novel mechanisms of myocardial pyroptosis and provides a new therapeutic target for septic shock-induced cardiac dysfunction.

## Data Availability

The original contributions presented in the study are included in the article/Supplementary Material, further inquiries can be directed to the corresponding authors.

## References

[B1] Adamo L. Rocha-Resende C. Prabhu S. D. Mann D. L. (2020). Reappraising the role of inflammation in heart failure. Nat. Rev. Cardiol. 17, 269–285. 10.1038/s41569-019-0315-x 31969688

[B2] Bartoli-Leonard F. Saddic L. Aikawa E. (2020). Double-edged sword of ALDH2 mutations: One polymorphism can both benefit and harm the cardiovascular system. Eur. Heart J. 41, 2453–2455. 10.1093/eurheartj/ehaa444 32585692 PMC7340353

[B3] Beesley S. J. Weber G. Sarge T. Nikravan S. Grissom C. K. Lanspa M. J. (2018). Septic cardiomyopathy. Crit. Care Med. 46, 625–634. 10.1097/CCM.0000000000002851 29227368

[B4] Bergsbaken T. Fink S. L. Cookson B. T. (2009). Pyroptosis: Host cell death and inflammation. Nat. Rev. Microbiol. 7, 99–109. 10.1038/nrmicro2070 19148178 PMC2910423

[B5] Broz P. Dixit V. M. (2016). Inflammasomes: Mechanism of assembly, regulation and signalling. Nat. Rev. Immunol. 16, 407–420. 10.1038/nri.2016.58 27291964

[B6] Carrico C. Meyer J. G. He W. Gibson B. W. Verdin E. (2018). The mitochondrial acylome emerges: Proteomics, regulation by sirtuins, and metabolic and disease implications. Cell Metab. 27, 497–512. 10.1016/j.cmet.2018.01.016 29514063 PMC5863732

[B7] Carriere J. Dorfleutner A. Stehlik C. (2021). NLRP7: From inflammasome regulation to human disease. Immunology 163, 363–376. 10.1111/imm.13372 34021586 PMC8274175

[B8] Chen C. H. Ferreira J. C. Gross E. R. Mochly-Rosen D. (2014). Targeting aldehyde dehydrogenase 2: New therapeutic opportunities. Physiol. Rev. 94, 1–34. 10.1152/physrev.00017.2013 24382882 PMC3929114

[B9] Chen X. Barozzi I. Termanini A. Prosperini E. Recchiuti A. Dalli J. (2012). Requirement for the histone deacetylase Hdac3 for the inflammatory gene expression program in macrophages. Proc. Natl. Acad. Sci. U. S. A. 109, E2865–E2874. 10.1073/pnas.1121131109 22802645 PMC3479529

[B10] Chi Z. Chen S. Xu T. Zhen W. Yu W. Jiang D. (2020). Histone deacetylase 3 couples mitochondria to drive IL-1β-dependent inflammation by configuring fatty acid oxidation. Mol. Cell 80, 43–58. 10.1016/j.molcel.2020.08.015 32937100

[B11] Del Re D. P. Amgalan D. Linkermann A. Liu Q. Kitsis R. N. (2019). Fundamental mechanisms of regulated cell death and implications for heart disease. Physiol. Rev. 99, 1765–1817. 10.1152/physrev.00022.2018 31364924 PMC6890986

[B12] Deng Y. Xie M. Li Q. Xu X. Ou W. Zhang Y. (2021). Targeting mitochondria-inflammation circuit by beta-hydroxybutyrate mitigates HFpEF. Circ. Res. 128, 232–245. 10.1161/CIRCRESAHA.120.317933 33176578

[B13] Dittenhafer-Reed K. E. Richards A. L. Fan J. Smallegan M. J. Fotuhi Siahpirani A. Kemmerer Z. A. (2015). SIRT3 mediates multi-tissue coupling for metabolic fuel switching. Cell Metab. 21, 637–646. 10.1016/j.cmet.2015.03.007 25863253 PMC4393847

[B14] Fenini G. Karakaya T. Hennig P. Di Filippo M. Beer H. D. (2020). The NLRP1 inflammasome in human skin and beyond. Int. J. Mol. Sci. 21, 4788. 10.3390/ijms21134788 32640751 PMC7370280

[B15] Fritsch M. Gunther S. D. Schwarzer R. Albert M. C. Schorn F. Werthenbach J. P. (2019). Caspase-8 is the molecular switch for apoptosis, necroptosis and pyroptosis. Nature 575, 683–687. 10.1038/s41586-019-1770-6 31748744

[B16] Gaul S. Leszczynska A. Alegre F. Kaufmann B. Johnson C. D. Adams L. A. (2021). Hepatocyte pyroptosis and release of inflammasome particles induce stellate cell activation and liver fibrosis. J. Hepatol. 74, 156–167. 10.1016/j.jhep.2020.07.041 32763266 PMC7749849

[B17] Gross E. R. Zambelli V. O. Small B. A. Ferreira J. C. Chen C. H. Mochly-Rosen D. (2015). A personalized medicine approach for Asian Americans with the aldehyde dehydrogenase 2*2 variant. Annu. Rev. Pharmacol. Toxicol. 55, 107–127. 10.1146/annurev-pharmtox-010814-124915 25292432 PMC4435945

[B18] He Y. Hara H. Nunez G. (2016). Mechanism and regulation of NLRP3 inflammasome activation. Trends Biochem. Sci. 41, 1012–1021. 10.1016/j.tibs.2016.09.002 27669650 PMC5123939

[B19] Hollenberg S. M. Singer M. (2021). Pathophysiology of sepsis-induced cardiomyopathy. Nat. Rev. Cardiol. 18, 424–434. 10.1038/s41569-020-00492-2 33473203

[B20] Houten S. M. Violante S. Ventura F. V. Wanders R. J. (2016). The biochemistry and Physiology of mitochondrial fatty acid beta-oxidation and its genetic disorders. Annu. Rev. Physiol. 78, 23–44. 10.1146/annurev-physiol-021115-105045 26474213

[B21] Huang L. S. Hong Z. Wu W. Xiong S. Zhong M. Gao X. (2020). mtDNA activates cGAS signaling and suppresses the YAP-mediated endothelial cell proliferation program to promote inflammatory injury. Immunity 52, 475–486. 10.1016/j.immuni.2020.02.002 32164878 PMC7266657

[B22] Jiang L. P. Yu X. H. Chen J. Z. Hu M. Zhang Y. K. Lin H. L. (2022). Histone deacetylase 3: A potential therapeutic target for atherosclerosis. Aging Dis. 13, 773–786. 10.14336/AD.2021.1116 35656103 PMC9116907

[B23] Jiang X. Cai S. Jin Y. Wu F. He J. Wu X. (2021). Irisin attenuates oxidative stress, mitochondrial dysfunction, and apoptosis in the H9C2 cellular model of septic cardiomyopathy through augmenting fundc1-dependent mitophagy. Oxid. Med. Cell Longev. 2021, 2989974. 10.1155/2021/2989974 34457111 PMC8390168

[B24] Jo E. K. Kim J. K. Shin D. M. Sasakawa C. (2016). Molecular mechanisms regulating NLRP3 inflammasome activation. Cell Mol. Immunol. 13, 148–159. 10.1038/cmi.2015.95 26549800 PMC4786634

[B25] Kang R. Zeng L. Zhu S. Xie Y. Liu J. Wen Q. (2018). Lipid peroxidation drives gasdermin D-mediated pyroptosis in lethal polymicrobial sepsis. Cell Host Microbe 24, 97–108. 10.1016/j.chom.2018.05.009 29937272 PMC6043361

[B26] Kempker J. A. Martin G. S. (2020). A global accounting of sepsis. Lancet 395, 168–170. 10.1016/S0140-6736(19)33065-X 31954445

[B27] Kovacs S. B. Miao E. A. (2017). Gasdermins: Effectors of pyroptosis. Trends Cell Biol. 27, 673–684. 10.1016/j.tcb.2017.05.005 28619472 PMC5565696

[B28] Li N. Zhou H. Wu H. Wu Q. Duan M. Deng W. (2019). STING-IRF3 contributes to lipopolysaccharide-induced cardiac dysfunction, inflammation, apoptosis and pyroptosis by activating NLRP3. Redox Biol. 24, 101215. 10.1016/j.redox.2019.101215 31121492 PMC6529775

[B29] Li Q. Hua X. Li L. Zhou X. Tian Y. Deng Y. (2022). AIP1 suppresses neovascularization by inhibiting the NOX4-induced NLRP3/NLRP6 imbalance in a murine corneal alkali burn model. Cell Commun. Signal 20, 59. 10.1186/s12964-022-00877-5 35524333 PMC9074213

[B30] Li Q. Zhang M. Zhao Y. Dong M. (2021). Irisin protects against LPS-stressed cardiac damage through inhibiting inflammation, apoptosis, and pyroptosis. Shock 56, 1009–1018. 10.1097/SHK.0000000000001775 34779800

[B31] Liu Y. Lu L. L. Wen D. Liu D. L. Dong L. L. Gao D. M. (2020). MiR-612 regulates invadopodia of hepatocellular carcinoma by HADHA-mediated lipid reprogramming. J. Hematol. Oncol. 13, 12. 10.1186/s13045-019-0841-3 32033570 PMC7006096

[B32] Man S. M. Kanneganti T. D. (2015). Gasdermin D: The long-awaited executioner of pyroptosis. Cell Res. 25, 1183–1184. 10.1038/cr.2015.124 26482951 PMC4650426

[B33] Mann D. L. (2015). Innate immunity and the failing heart: The cytokine hypothesis revisited. Circ. Res. 116, 1254–1268. 10.1161/CIRCRESAHA.116.302317 25814686 PMC4380242

[B34] Merx M. W. Weber C. (2007). Sepsis and the heart. Circulation 116, 793–802. 10.1161/CIRCULATIONAHA.106.678359 17698745

[B35] Miklas J. W. Clark E. Levy S. Detraux D. Leonard A. Beussman K. (2019). TFPa/HADHA is required for fatty acid beta-oxidation and cardiolipin re-modeling in human cardiomyocytes. Nat. Commun. 10, 4671. 10.1038/s41467-019-12482-1 31604922 PMC6789043

[B36] Nguyen H. C. B. Adlanmerini M. Hauck A. K. Lazar M. A. (2020). Dichotomous engagement of HDAC3 activity governs inflammatory responses. Nature 584, 286–290. 10.1038/s41586-020-2576-2 32760002 PMC7725280

[B37] Olsen M. B. Gregersen I. Sandanger O. Yang K. Sokolova M. Halvorsen B. E. (2022). Targeting the inflammasome in cardiovascular disease. JACC Basic Transl. Sci. 7, 84–98. 10.1016/j.jacbts.2021.08.006 35128212 PMC8807732

[B38] Pang J. Peng H. Wang S. Xu X. Xu F. Wang Q. (2019). Mitochondrial ALDH2 protects against lipopolysaccharide-induced myocardial contractile dysfunction by suppression of ER stress and autophagy. Biochim. Biophys. Acta Mol. Basis Dis. 1865, 1627–1641. 10.1016/j.bbadis.2019.03.015 30946956

[B39] Panov A. Mayorov V. I. Dikalov S. (2022). Metabolic syndrome and beta-oxidation of long-chain fatty acids in the brain, heart, and kidney mitochondria. Int. J. Mol. Sci. 23, 4047. 10.3390/ijms23074047 35409406 PMC9000033

[B40] Parrillo J. E. Parker M. M. Natanson C. Suffredini A. F. Danner R. L. Cunnion R. E. (1990). Septic shock in humans. Advances in the understanding of pathogenesis, cardiovascular dysfunction, and therapy. Ann. Intern Med. 113, 227–242. 10.7326/0003-4819-113-3-227 2197912

[B41] Pulido J. N. Afessa B. Masaki M. Yuasa T. Gillespie S. Herasevich V. (2012). Clinical spectrum, frequency, and significance of myocardial dysfunction in severe sepsis and septic shock. Mayo Clin. Proc. 87, 620–628. 10.1016/j.mayocp.2012.01.018 22683055 PMC3538477

[B42] Qiao L. Ma J. Zhang Z. Sui W. Zhai C. Xu D. (2021). Deficient chaperone-mediated autophagy promotes inflammation and atherosclerosis. Circ. Res. 129, 1141–1157. 10.1161/CIRCRESAHA.121.318908 34704457 PMC8638823

[B43] Rathinam V. A. Fitzgerald K. A. (2016). Inflammasome complexes: Emerging mechanisms and effector functions. Cell 165, 792–800. 10.1016/j.cell.2016.03.046 27153493 PMC5503689

[B44] Rathkey J. K. Zhao J. Liu Z. Chen Y. Yang J. Kondolf H. C. (2018). Chemical disruption of the pyroptotic pore-forming protein gasdermin D inhibits inflammatory cell death and sepsis. Sci. Immunol. 3, eaat2738. 10.1126/sciimmunol.aat2738 30143556 PMC6462819

[B45] Rudd K. E. Johnson S. C. Agesa K. M. Shackelford K. A. Tsoi D. Kievlan D. R. (2020). Global, regional, and national sepsis incidence and mortality, 1990-2017: Analysis for the global burden of disease study. Lancet 395, 200–211. 10.1016/S0140-6736(19)32989-7 31954465 PMC6970225

[B46] Shi J. Gao W. Shao F. (2017). Pyroptosis: Gasdermin-Mediated programmed necrotic cell death. Trends Biochem. Sci. 42, 245–254. 10.1016/j.tibs.2016.10.004 27932073

[B47] Shrum B. Anantha R. V. Xu S. X. Donnelly M. Haeryfar S. M. Mccormick J. K. (2014). A robust scoring system to evaluate sepsis severity in an animal model. BMC Res. Notes 7, 233. 10.1186/1756-0500-7-233 24725742 PMC4022086

[B48] Singer M. Deutschman C. S. Seymour C. W. Shankar-Hari M. Annane D. Bauer M. (2016). The third international consensus definitions for sepsis and septic shock (Sepsis-3). JAMA 315, 801–810. 10.1001/jama.2016.0287 26903338 PMC4968574

[B49] Sulzbacher M. M. Sulzbacher L. M. Passos F. R. Bilibio B. L. E. Althaus W. F. Weizenmann L. (2020). A single dose of eHSP72 attenuates sepsis severity in mice. Sci. Rep. 10, 9198. 10.1038/s41598-020-66011-y 32513986 PMC7280184

[B50] Swanson K. V. Deng M. Ting J. P. (2019). The NLRP3 inflammasome: Molecular activation and regulation to therapeutics. Nat. Rev. Immunol. 19, 477–489. 10.1038/s41577-019-0165-0 31036962 PMC7807242

[B51] Wang J. Wang H. Hao P. Xue L. Wei S. Zhang Y. (2011). Inhibition of aldehyde dehydrogenase 2 by oxidative stress is associated with cardiac dysfunction in diabetic rats. Mol. Med. 17, 172–179. 10.2119/molmed.2010.00114 20957334 PMC3060979

[B52] Wang X. Li X. Liu S. Brickell A. N. Zhang J. Wu Z. (2020). PCSK9 regulates pyroptosis via mtDNA damage in chronic myocardial ischemia. Basic Res. Cardiol. 115, 66. 10.1007/s00395-020-00832-w 33180196

[B53] Xiong X. Lu L. Wang Z. Ma J. Shao Y. Liu Y. (2022). Irisin attenuates sepsis-induced cardiac dysfunction by attenuating inflammation-induced pyroptosis through a mitochondrial ubiquitin ligase-dependent mechanism. Biomed. Pharmacother. 152, 113199. 10.1016/j.biopha.2022.113199 35653888

[B54] Xu T. Sun L. Shen X. Chen Y. Yin Y. Zhang J. (2019). NADPH oxidase 2-mediated NLRP1 inflammasome activation involves in neuronal senescence in hippocampal neurons *in vitro* . Int. Immunopharmacol. 69, 60–70. 10.1016/j.intimp.2019.01.025 30677569

[B55] Xue Y. Enosi Tuipulotu D. Tan W. H. Kay C. Man S. M. (2019). Emerging activators and regulators of inflammasomes and pyroptosis. Trends Immunol. 40, 1035–1052. 10.1016/j.it.2019.09.005 31662274

[B56] Zhang Y. Huang Z. Li H. (2017). Insights into innate immune signalling in controlling cardiac remodelling. Cardiovasc Res. 113, 1538–1550. 10.1093/cvr/cvx130 29088374

[B57] Zheng M. Karki R. Vogel P. Kanneganti T. D. (2020). Caspase-6 is a key regulator of innate immunity, inflammasome activation, and host defense. Cell 181, 674–687. 10.1016/j.cell.2020.03.040 32298652 PMC7425208

[B58] Zhong S. Li L. Zhang Y. L. Zhang L. Lu J. Guo S. (2019). Acetaldehyde dehydrogenase 2 interactions with LDLR and AMPK regulate foam cell formation. J. Clin. Invest. 129, 252–267. 10.1172/JCI122064 30375985 PMC6307968

[B59] Zhong Z. Liang S. Sanchez-Lopez E. He F. Shalapour S. Lin X. J. (2018). New mitochondrial DNA synthesis enables NLRP3 inflammasome activation. Nature 560, 198–203. 10.1038/s41586-018-0372-z 30046112 PMC6329306

[B60] Zhong Z. Umemura A. Sanchez-Lopez E. Liang S. Shalapour S. Wong J. (2016). NF-κB restricts inflammasome activation via elimination of damaged mitochondria. Cell 164, 896–910. 10.1016/j.cell.2015.12.057 26919428 PMC4769378

[B61] Zhou R. Yazdi A. S. Menu P. Tschopp J. (2011). A role for mitochondria in NLRP3 inflammasome activation. Nature 469, 221–225. 10.1038/nature09663 21124315

