# Supplementary material for: ALDH2 attenuates myocardial pyroptosis through breaking down Mitochondrion-NLRP3 inflammasome pathway in septic shock
Source: Front Pharmacol. 2023 Mar 13;14:1125866. doi: 10.3389/fphar.2023.1125866 (PMC10040788; doi:10.3389/fphar.2023.1125866)
Supplement: Supplementary file 1 [file DataSheet1.pdf]

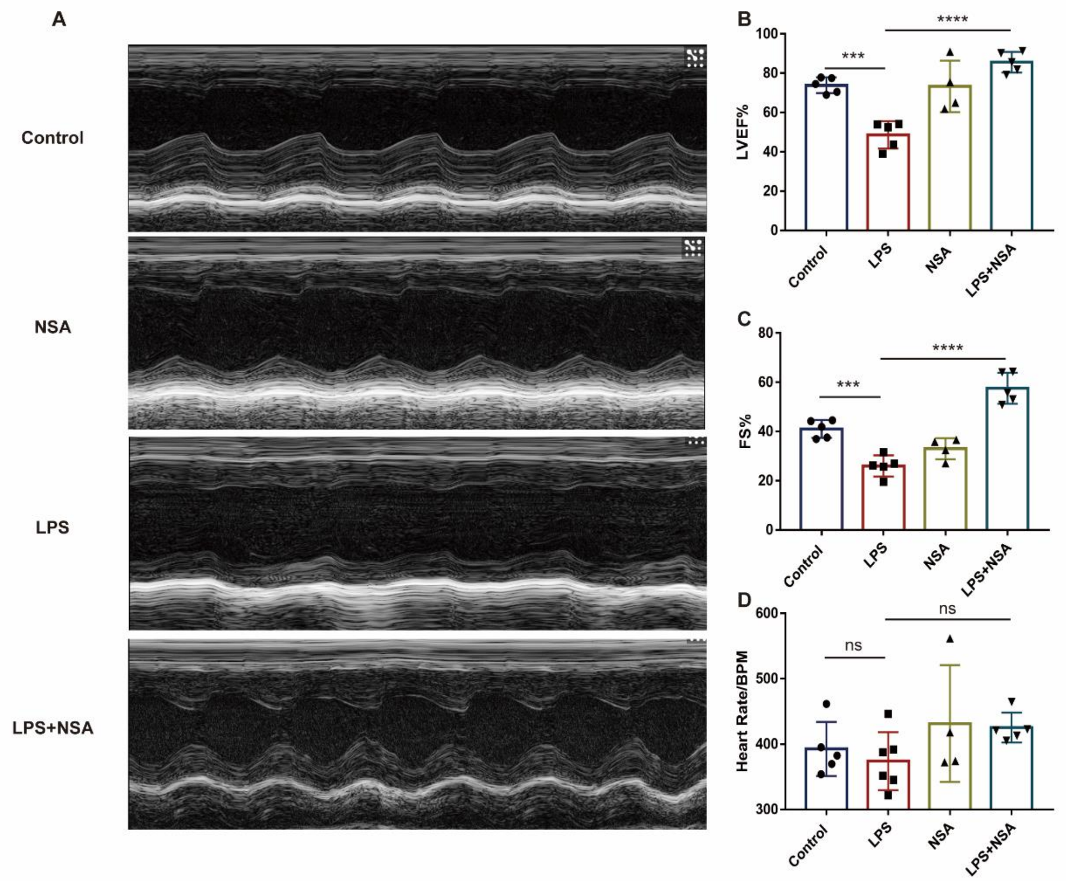

Supplementary Figure 1. Role of pyroptosis in LPS-induced cardiac dysfunction. C57BL/6 mice were treated with or without LPS (15 mg/kg, 12 hrs) in the absence or presence of the pyroptosis inhibitor NSA (20mg/kg). **(A)** Representative echocardiographic images from four mouse groups. **(B-D)** Quantification of the echocardiography. n=4-5, Mean ± SEM. ****$P <$ 0.0001; ***$P <$ 0.001; **$P <$ 0.01; *$P <$ 0.05; ns=not significant.

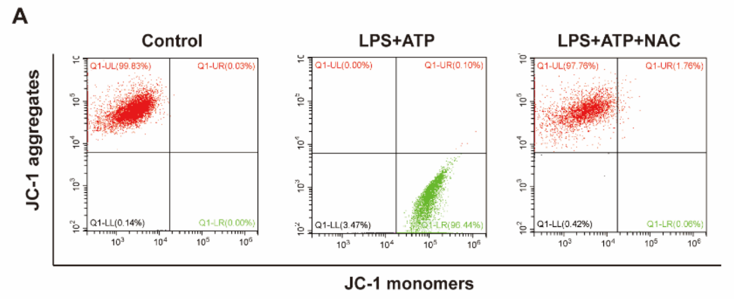

Supplementary Figure 2. The effect of ALDH2 on mitochondrial membrane potential in cardiomyocytes. (A) Representative images of JC-1 staining in LPS plus ATP-simulated or control H9C2 cells with or without pre-treated NAC (50μmol/L, 1 hrs), Mean ± SEM. ****P < 0.0001; ***P < 0.001; **P < 0.01; *P < 0.05; ns=not significant.
